# CD4 and CD8 T-cell response is dominated by IL-10–secreting cells in children with uncomplicated *Plasmodium falciparum* malaria

**DOI:** 10.1093/immhor/vlaf045

**Published:** 2025-11-24

**Authors:** Bonface O Ariera, Bernard Guyah, Ian Onditi, Kevin Waomba, Emily Koech, Katherine R Sabourin, Gabriela Samayoa-Reyes, Rosemary Rochford, Sidney Ogolla

**Affiliations:** Department of Biomedical Sciences and Technology, Maseno University, Maseno, Kenya; Center for Global Health Research, Kenya Medical Research Institute, Kisumu, Kenya; Department of Biomedical Sciences and Technology, Maseno University, Maseno, Kenya; Center for Global Health Research, Kenya Medical Research Institute, Kisumu, Kenya; Center for Global Health Research, Kenya Medical Research Institute, Kisumu, Kenya; Center for Global Health Research, Kenya Medical Research Institute, Kisumu, Kenya; Department of Environmental and Public Health Sciences, University of Cincinnati, Cincinnati, OH, United States; Vaccine and Gene Therapy Institute, Oregon Health and Science University, Portland, OR, United States; Department of Immunology and Microbiology, University of Colorado, Anschutz Medical Campus, Aurora, CO, United States; Center for Global Health Research, Kenya Medical Research Institute, Kisumu, Kenya

**Keywords:** EBV, malaria, Burkitt lymphoma, T-cell immunity

## Abstract

*Plasmodium falciparum* malaria and Epstein-Barr virus (EBV) coinfections have been associated with an increased risk of developing the EBV-associated cancer endemic Burkitt lymphoma (eBL). In children living in malaria-endemic areas, repeated episodes of malaria may alter the immune system’s ability to suppress EBV, creating a permissive environment for eBL pathogenesis. However, the malaria-driven mechanisms involved remain undefined, including whether malaria-induced immune alterations are EBV-specific or systemic. To identify whether acute clinical *P. falciparum* malaria affects EBV T-cell immunity, we characterized T-cell activation status and cytokine secretion profiles using flow cytometry. We compared profiles in 10 Kenyan children with acute clinical *P. falciparum* malaria at baseline and matched 4-week recovery, and 10 healthy community controls following antigenic stimulation with EBV- and cytomegalovirus-specific peptides. The percentage frequency of activation-induced marker cells was comparable within the study cohort across the different stimulations. Furthermore, we observed a shift in cytokine secretion in children with acute malaria during active disease and at 4 weeks postrecovery, favoring IL-10 for both T-cell subsets. Our findings suggest that clinical malaria did not result in the impairment of T-cell activation, but rather induced shifts in cytokine secretion in favor of IL-10. We further demonstrate that malaria-induced T-cell immune alterations are not EBV-specific but rather affect overall immune suppression.

## Introduction

Since its discovery in 1958, endemic Burkitt lymphoma (eBL) remains one of the prevalent childhood cancers in sub-Saharan Africa.^1^^,^^2^ The increased risk for this lymphoma is linked to coinfections with *Plasmodium falciparum* malaria and Epstein-Barr virus (EBV).^3–5^ Early age of primary EBV infection coupled with repeated *P. falciparum* malaria is associated with poor viral control and thus increased risk of eBL development.^6^

EBV is a lifelong gammaherpesvirus that persistently infects up to 95% of the global population.^7^ EBV has evolved to coexist in humans by latently infecting B cells in the majority of people without serious complications.^7^^,^^8^ However, the virus is linked to malignant transformation of infected cells as in the case of eBL^8^. Infection with EBV elicits both cellular and humoral immune responses. The serological response to EBV is well characterized and has been used clinically to identify primary, past, or reactivated viral infection.^9^ Individuals infected with EBV present with a diverse repertoire of neutralizing antibodies that target EBV-encoded proteins.^10^ A number of studies have described the effects of both acute and chronic *P. falciparum* malaria on EBV infection.^11–14^ Evidence from previous studies suggests that *P. falciparum* malaria induces EBV reactivation among children living in malaria-holoendemic areas, consequently resulting in elevated EBV DNA and EBV-specific IgG antibodies.^15^ Elevation of antibodies to the EBV viral capsid antigen (VCA) were found to precede the emergence of eBL,^16^^,^^17^ and furthermore, the inability of lymphocytes from malaria-infected individuals to control the proliferation of EBV-infected B cells suggests either loss of EBV immunity or lytic reactivation of the virus or both.^18^^,^^19^

Control of EBV infections is primarily mediated by a human leukocyte antigen class I–restricted cytotoxic CD8^+^ T lymphocyte (CTL) response.^20^ In the absence of an effective CTL response in immunocompromised patients, increased EBV viral load in the peripheral blood indicates a key link between CTL function and EBV persistence. Several studies have found that *P. falciparum* malaria impairs EBV-specific T-cell responses.^5^^,^^12^^,^^13^^,^^21–23^ Because EBV is a strict human pathogen, addressing the role of malaria in affecting EBV-specific immunity has relied on 2 different study designs. In one approach, studies compared adults and children living in malaria-holoendemic regions to individuals residing in the highlands with low malaria prevalence and found that those in the holoendemic regions had impaired EBV-specific T-cell responses.^10^^,^^11^ In a second approach, a comparison of EBV immune responses in adults or children with malaria was done.^12^^,^^13^ For example, peripheral blood lymphocytes isolated from adult patients with acute *P. falciparum* malaria were unable to control the outgrowth of EBV-transformed cells.^13^ Njie et al. showed that adults with acute malaria did not have a suppressed IFN-γ response, while among 4 children with acute malaria, they observed weak IFN-γ responses that recovered at 4 weeks posttreatment.^12^ Collectively, these studies provide support for a malaria-induced suppression of EBV T-cell immunity that is age-specific, either through loss of EBV-specific T cells or through functional impairment of circulating T cells.

A more direct look at EBV-specific immunity deficit involved eBL patients.^24^ Using overlapping peptide pools to EBV nuclear antigen 1 (EBNA-1) proteins, children with eBL were found to have significantly reduced frequency of EBV-specific T cells and in those who did respond, the magnitude of the response was also diminished compared to age-matched children without eBL.^24^ Interestingly, in a follow-up study, eBL patients were shown to have more CD4^+^ EBNA-1–specific IL-10 T-cell responses compared to age-matched children without eBL.^24^ Beyond these earlier studies that informed the theory that EBV-specific T-cell responses are selectively altered during malaria coinfection using the crude but functional regression assays or the more sensitive ELISPOT assay, studies into the mechanism by which *P. falciparum* malaria might potentiate EBV-specific T-cell immunity deficit are limited. It is established that the malaria parasite modulates the host immune system.^25–27^ For example, in murine models, *Plasmodium* inhibits dendritic cell maturation with the consequence of suppression of IFN-γ–secreting CD8 T cells^28^ and the potential for inducing regulatory CD8 T cells that express IL-10.^18^ We found a strong inverse correlation between IL-10 production by EBV-specific CD8 T cells and cytotoxic function, suggesting a link between malaria-mediated immunosuppression and control of EBV-infected cells.^29^ Although well studied in other infections, the surface markers and functional characteristics of CD4 and CD8 cells have not been examined in detail in the context of EBV and malaria coinfections.

The advent of the activation-induced marker (AIM) assay has allowed for the comprehensive analysis of distinct immune cell subsets and identification of changes induced by the infection.^30^^,^^31^ This approach allows for a granularity of understanding of subset-specific cell responses following antigenic stimulation, not previously possible with the traditional methods. Given the importance of defining the transforming events mediated by *P. falciparum* malaria and EBV coinfection on eBL pathogenesis, we used a flow cytometry AIM assay that allows for simultaneous detection and phenotypic characterization of EBV-specific CD4 and CD8 T-cell responses.^32^ By virtue of the highly multiplex nature of the AIM assay, we defined both CD4^+^ and CD8^+^ antigen-specific T-cell responses as well as cytokine secretion profiles. We included an evaluation of CD4 and CD8 T-cell responses to human cytomegalovirus (CMV), another chronic viral infection, to ask whether the reported malaria-induced immune perturbations were EBV-specific or reflected a more global suppression. Accordingly, we observed altered frequencies in T-cell phenotypes during uncomplicated malaria infection. Further, our results from the T-cell activation and cytokine secretory profiles led us to propose that a malaria-induced shift from the Th1 IFN-γ response to a regulatory T cell–based IL-10 response is one possible mechanism for the loss of control of EBV in eBL etiology.

## Materials and methods

### Study population

The study cohort consisted of children with uncomplicated *P. falciparum* malaria, defined by the presence of fever of >37.5 °C and positive for malaria parasites by microscopy and rapid diagnostic test (RDT), who were enrolled at the Chulaimbo Sub-County Hospital in western Kenya. Healthy age-matched children (herein referred to as community controls) with no history of clinical malaria–related signs and symptoms (as reported by guardians) were screened for *P. falciparum* malaria by RDT and microscopy and enrolled as controls. Children with uncomplicated malaria were sampled during clinical disease, and additional samples were collected at 4 weeks after initiation of treatment (4-week recovery). Malaria infection status in the study cohorts was further confirmed by quantitative polymerase chain reaction (qPCR) as previously described.^11^

Written informed consent was obtained from the parents/guardians before the children’s enrollment in the study. Ethical approval was obtained from the Colorado Multiple Institutional Review Board at the University of Colorado, Anschutz Medical Campus, Aurora, Colorado, United States, and the Scientific and Ethics Review Unit at the Kenya Medical Research Institute, Nairobi, Kenya.

### T-cell responses by activation-induced markers and flow cytometry staining

PBMCs previously collected and cryopreserved in freezing media consisting of 90% FBS (Cell Generation) and 10% DMSO (Sigma-Aldrich) cells were thawed, washed, and rested for 24 hours in warm 10 mL cRPMI (RPMI [Gibco] supplemented with 10% FBS [Cell Generation] and 1% penicillin-streptomycin and 1% glutamine [Gibco]) at 37 °C in a 5% CO_2_ incubator. After the rest, cells were stimulated for 24 hours with the peptide pools. For each participant, 5 × 10^5^ PBMCs were separately stimulated with 5 μg/mL synthetic lytic and latent EBV peptides, including 15-mers overlapping by 11 amino acids spanning the whole BZLF1 protein sequence (59 peptides) and 15-mers overlapping by 11 amino acids spanning the immunogenic C-terminal region of EBNA-1 protein (279 peptides). Peptides with a purity of ≥95% were purchased from JPT Peptides Technologies GmbH and Stemcell Technologies for BZLF1 and EBNA-1, respectively. A mock negative control (DMSO) and positive control with CMV p65 (Stemcell Technologies) were included for each participant. The cells were incubated at 37 °C in a 5% CO_2_ incubator. To inhibit cytokine secretion, Brefeldin A (Sigma-Aldrich) at a concentration of 1 μg/mL was added to the stimulation for 4 hours toward the end of incubation. Cells were harvested, washed in FACS buffer (PBS supplemented with 2% FBS), and incubated with a cell surface antibody cocktail (Table S1). Following incubation for 30 minutes at 4 °C, cells were washed once in FACS buffer, fixed, and permeabilized for 30 minutes at room temperature (Foxp3/Transcription Factor Fixation/Permeabilization Concentrate and Diluent, eBioscience), and washed once in 1× permeabilization buffer prior to intracellular staining. Cells were then incubated with an intracellular antibody cocktail of anti-IL-10 and anti-IFN-γ. Cells were then washed once in 1× permeabilization buffer and resuspended in FACS wash prior to data acquisition on a 5L Cytek Aurora flow cytometer.

### EBV and *P. falciparum* microsphere immunoassay

Plasma from the study subjects were tested for IgG antibody levels against recombinant EBNA-1 and VCA, as previously described,^33^ with subtle modifications. Malaria antibody levels against apical membrane protein 1 (AMA-1), merozoite surface protein 1 (MSP-1), and circumsporozoite surface protein (CSP) antigens were also tested in a similar way. In brief, plasma was diluted at 1:200 and incubated with pooled bead conjugates for 1 hour at room temperature on a plate shaker at 800 rpm in the dark. Plates were then put on a magnetic separator and washed twice with PBS-TBN buffer. Sample were then incubated with anti-human IgG-PE detection antibody (R&D Systems) for 2 hours on a plate shaker at the same speed. Samples were washed twice and then resuspended in 100 μL of the PBS-TBN buffer. The median fluorescence intensity (MFI) was then obtained from the MagPix XMap Luminex machine. Three negative control plasma samples from known EBV- and malaria-negative U.S. donors were included. In addition, a pool of EBV^+^ plasma from Kenyan adult donors was used a positive control. Seropositivity cutoff was determined as the mean MFI plus 3 times the SD of the U.S. donor samples for each antigen.

### *Plasmodium falciparum* and EBV qPCR

*P. falciparum* DNA was extracted from 200 μL of whole blood obtained via a finger prick into EDTA tubes (BD Vacutainer) using the Qiagen DNAeasy kit according to the manufacturer’s protocol. *P. falciparum* was quantified using the primer set targeting the 18S as previously described^34^ on a ViiA7 machine (AB Applied Biosystems by Life Technologies). *P. falciparum* plasmid of known concentration was used for the quantification of *P. falciparum*, and data are expressed as copies/mL.

The EBV DNA was quantified by qPCR as described previously^6^^,^^35^ using the EBV BALF5 gene as target and the β-actin gene as control for DNA input. The limit of detection is 2 copies EBV/μg. The EBV load was expressed as copies/µg of DNA.

### Statistical analysis

Flow cytometry data were analyzed with FlowJo software, version 10.9.1 (BD), and statistics were performed using GraphPad Prism software, version 10.1.0. Boolean gating was used to characterize and determine T-cell functionality. All tests were 2-tailed and *P < *0.05 was set as the level of significance. All of the tests used are indicated in the legend of each figure or table. Among the children with uncomplicated malaria, baseline and 4-week postrecovery results were compared using the Wilcoxon signed-rank test. Community controls were compared to uncomplicated malaria (baseline or 4-week recovery) by the Mann–Whitney test. Results were expressed using the mean with SD for the dot plots and the exact *P* values.

## Results

### Study participants’ demographic characteristics

Children with uncomplicated *P. falciparum* malaria were recruited at the clinics of Chulaimbo Sub-County Hospital located in a rural area of Kisumu county, western Kenya, a region known for *P. falciparum* malaria holoendemicity. Our community controls were also obtained from the same rural community within a 10-km radius from the study hospital. The clinical and demographic characteristics are presented in Table 1. We analyzed samples from 10 children with uncomplicated *P. falciparum* malaria during active disease and at 4 weeks postrecovery and 10 community controls. The median age was 3.2 years (interquartile range [IQR], 2.5 to 4.4 years) and 3.8 years (IQR, 1.7 to 4.6 years) for children with uncomplicated malaria and community controls, respectively. The children were sex matched, with 50% (*n* = 5) males and 50% (*n* = 5) females for both the cases and community controls. To evaluate evidence of past infection with malaria and EBV, all of the children within the study cohort were seropositive for malaria and EBV by at least one of the antibodies tested. Community controls had no detected EBV virus and *P. falciparum* parasites by qPCR. Of the 10 children with uncomplicated malaria, we had 8 paired samples that were tested for EBV and *P. falciparum* load based on the availability of DNA samples. All 8 samples were positive for *P. falciparum* during active disease and at recovery (Fig. S1A), and 6 samples had EBV loads detectable by both timepoints (Fig. S1B).

**Table 1.** Demographics and clinical characteristics.

Characteristic	Acute	4 weeks postrecovery	Community controls
	(*n* = 10)	(*n* = 10)	(*n* = 10)
**Age, yr, median (IQR)**	3.2 (2.5–4.4)	3.2 (2.5–4.4)	3.8 (1.7–4.6)
**Sex, female**	5 (50.0%)	5 (50.0%)	5 (50.0%)
**Hemoglobin, g/dL, median (IQR)**	10.2 (9.4–10.9)	10.8 (10.2–11.3)	11.8 (10.4–12.6)
***Plasmodium* malaria**			
**Parasite load (copies/mL), median (IQR)**	7.3150e + 09 (1.5850e + 09–1.4125e + 10)	13,400 (5,490–236,750)	–
**EBV load (copies/µg), median (IQR)**	14,139.6 (1,599.5–58,296.4)	3,159.2 (1,228.8–5,637.3)	–

### Comparable levels of *P. falciparum* malaria and EBV antibodies in children with uncomplicated malaria and community controls

**Figure 1.**
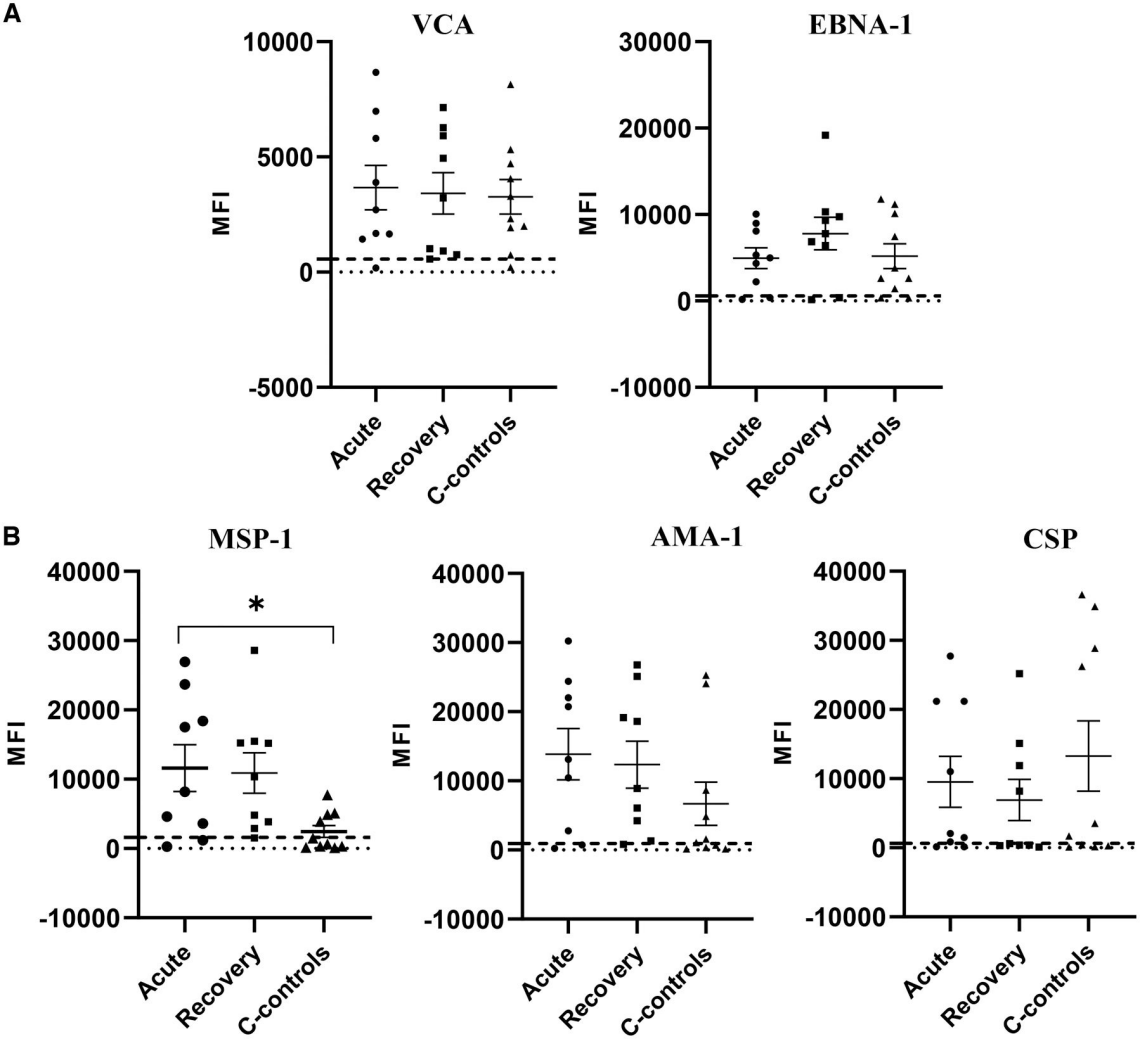
EBV- and *P. falciparum*–specific IgG antibodies in children with uncomplicated malaria, at 4 weeks postrecovery, and community controls. Plasma was diluted at 1:200 and tested using Luminex bead-based assay. The MFI of 50 beads for each antigen is indicated on the *y* axis. (A and B) MFI levels for EBV antigens (A) and for the different malaria antigens (B). A *P* value <0.05 was considered significant. *P < 0.04.

As a prelude to the T-cell antigen-specific immunity assays in the study group, we compared IgG antibody levels to EBV (EBNA-1 and VCA) and *P. falciparum* malaria (MSP-1, AMA-1, and CSP) antigens between children with uncomplicated malaria and community controls. This was important because all of the children in our study groups were from a malaria-holoendemic region and thereby likely to have experienced clinical/subclinical malaria episodes before enrollment in the study. Table 2 summarizes the median and IQRs of the MFI of IgG specific for the different EBV and *P. falciparum* malaria antigens tested. Remarkably, the median MFI levels of IgG against EBV EBNA-1 and VCA were comparable between children with uncomplicated malaria during active disease, at 4-week recovery, and community controls (Fig. 1A).

**Table 2.** Median levels and interquartile range of IgG-specific antibodies for different antigens.

Group	VCA	EBNA-1	AMA-1	MSP-1	CSP
**Acute**	2,323 (1,937–4,051)	4,998 (2,222–8,084)	13,086 (2,789–22,041)	8,197 (3,607–18,394)	2,044 (852–21,167)
**Recovery**	3,223 (918–5,922)	7,776 (6,428–9,740)	8,894 (4,241–19,162)	10,352 (3,857–15,209)	612 (381–11,876)
**Controls**	3,305 (1,659–6,685)	3,217 (1,732–9,445)	1,305 (443–7,686)	1,053 (304–4,631)	2,593 (322–28,197)

Next, we compared anti-AMA-1, CSP, and MSP-1 IgG-MFI levels in children with uncomplicated malaria during an active episode and at recovery, and in the community controls. There were no significant differences in the levels of antibodies against CPS and AMA-1 between the study groups (Fig. 1B). The levels of MSP-1 antibodies were significantly elevated in children with uncomplicated malaria during active disease compared to community controls (Fig. 1B, *P *= 0.04). There were no differences in the level of anti-MSP-1 antibodies in children at recovery and community controls (Fig. 1B).

### Altered frequency of CD4^+^ and CD8^+^ T-cell subsets in children with uncomplicated malaria and community controls

Since uncomplicated malaria has previously been reported to alter lymphocyte subpopulations,^36–38^ we compared the percentage frequency of total CD3^+^, CD4^+^, and CD8^+^ T-cell subsets in our study groups. While we observed no significant difference in the percentages of total CD3^+^ cells between children with uncomplicated malaria during active disease, at 4 weeks postrecovery, and community controls, CD4^+^ T-cell percentage was significantly reduced during uncomplicated malaria as compared to 4 weeks postrecovery (60.8% vs 71.4%, Table 3). In contrast, though not statistically significant, the percentage frequency of CD8^+^ T cells was elevated during uncomplicated malaria compared to 4 weeks postrecovery (28.6% vs 22.2%) and community controls (28.6% vs 23.5%) (Table 3).

**Table 3.** Mean percentage of lymphocyte subsets during uncomplicated malaria, recovery, and community controls.

Cell type	Acute	4 weeks postrecovery	Community controls	*P* value
	Mean (SEM), %	Mean (SEM), %	Mean (SEM), %	A vs R; A vs C; R vs C
**CD3^+^**	78.4 (3.0)	80.7 (2.4)	78.6 (2.6)	0.55; 0.94; 0.56
**CD4^+^**	60.8 (2.7)	71.4 (2.8)	68.2 (3.4)	**0.01**; 0.10; 0.45
**CD8^+^**	28.6 (2.0)	22.2 (2.6)	23.5 (1.8)	0.07; 0.08; 0.68
**CD4^+^ EM**	7.2 (1.2)	5.7 (0.9)	6.1 (1.1)	0.34; 0.50; 0.800
**CD4^+^ CM**	10.7 (1.0)	9.4 (1.1)	13.7 (1.6)	0.40; 0.13; **0.040**
**CD4^+^ TEMRA**	1.8 (0.4)	1.6 (0.2)	0.7 (0.1)	0.68; **0.01**; **0.003**
**CD4^+^ Naive**	80.3 (2.1)	83.2 (1.9)	79.5 (2.7)	0.32; 0.82; 0.270
**CD8^+^ EM**	4.9 (1.0)	4.4 (0.6)	7.3 (1.3)	0.66; 0.18; 0.08
**CD8^+^ CM**	1.3 (0.2)	1.6 (0.4)	2.4 (0.3)	0.50; **0.01**; 0.11
**CD8^+^ TEMRA**	16.7 (4.0)	11 (1.9)	14.6 (2.3)	0.22; 0.67; 0.24
**CD8^+^ Naive**	77.2 (4.6)	83 (1.6)	75.7 (3.5)	0.25; 0.81; 0.08

Significant differences are in bold.

**Figure 2.**
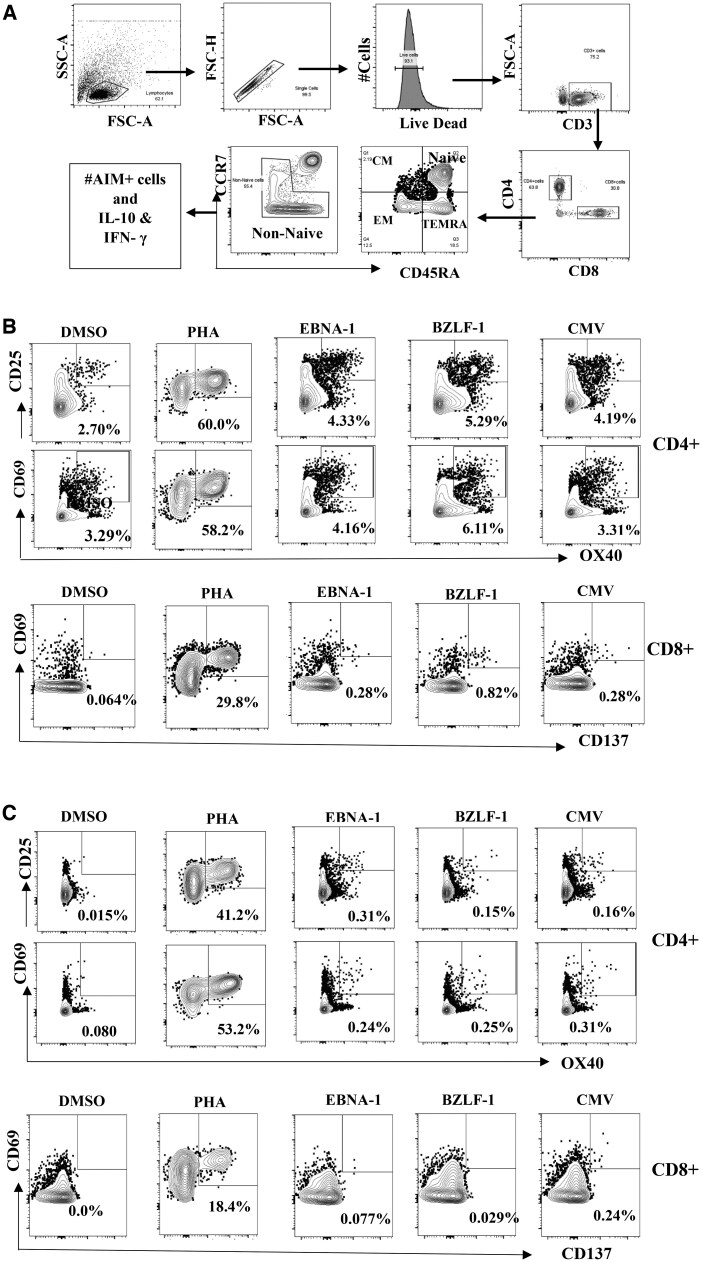
Flow cytometry gating on T-cell subsets and AIM^+^ cells. (A) After gating on the lymphocytes via SSC-A/FSC-A cytograms, singlets were selected by FSC-H/FSC-A. Live/Dead staining with Zombie Aqua allowed for selection of only live cells. CD3^+^ cells were gated by FSC/CD3 gating followed by CD4/CD8 subset gating. T-cell subsets were then defined by CD45RA/CCR7. AIM^+^ cells and cytokines were gated from the nonnaïve cells. (B and C) Representative gating on AIM^+^ cells in healthy Kenyan adult donors (B) and malaria-naïve U.S. donors (C). PHA was included as positive control.

We further characterized CD4^+^ and CD8^+^ into 4 subsets based on the expression of CD45RA and CCR7 (see Fig. 2 for the gating strategy). CD4^+^ and CD8^+^ T-cell subsets were defined as follows: naïve, CCR7^+^CD45RA; central memory (CM), CCR7^+^CD45RA^−^; effector memory (EM), CCR7^−^CD45RA^−^; and terminal effector memory (TEMRA), CCR7^−^CD45RA^+^. Children with uncomplicated malaria at 4 weeks of recovery had lower levels of CM CD4^+^CD45RA^−^CCR7^+^ compared to community controls (9.4% vs 13.7%). Children with uncomplicated malaria and 4-week recovery had elevated levels of TEMRA CD4^+^CD45RA^+^CCR7^−^ versus community controls (1.8% vs 0.7% and 1.6% vs 0.7%, respectively). For CD8^+^, the percentage frequency of the CM (CD8^+^CD45RA^−^CCR7^+^) subset was significantly reduced during uncomplicated malaria compared to 4 weeks postrecovery (1.3% vs 1.6%, Table 3).

### Similar T-cell activation profiles in children with uncomplicated malaria and community controls

**Figure 3.**
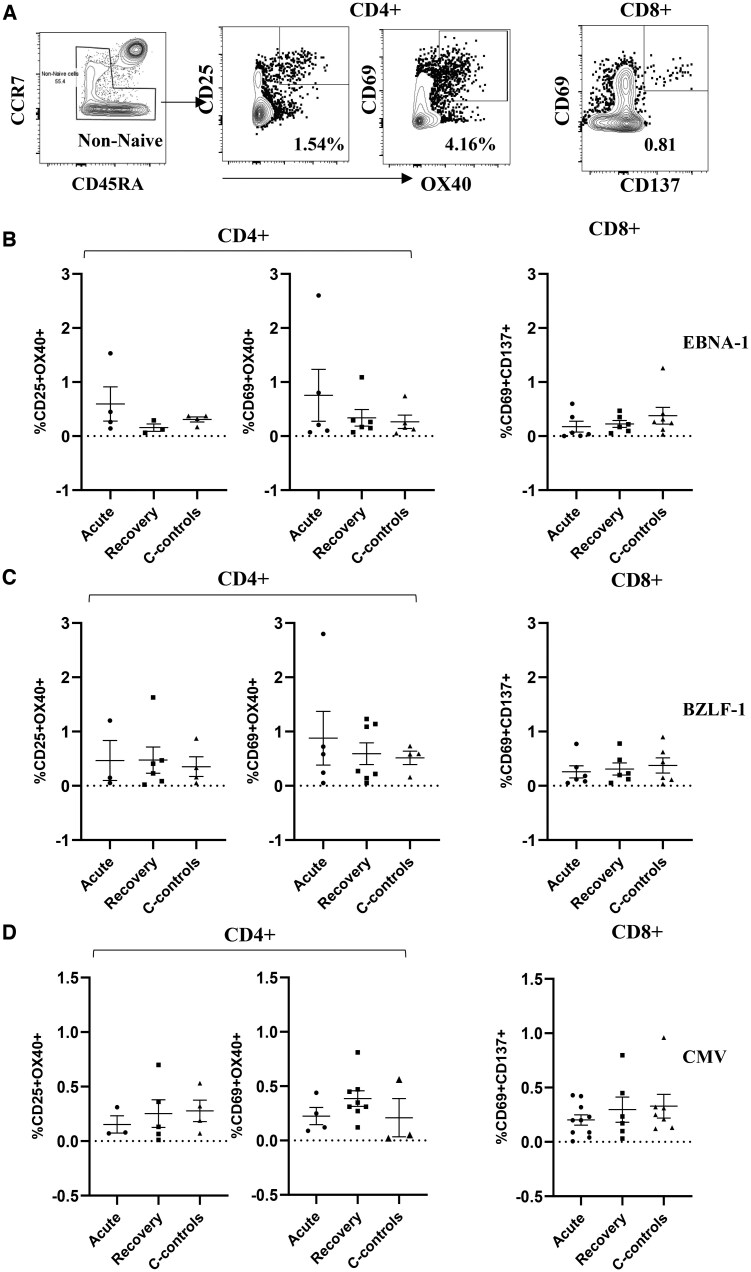
Comparison of AIM^+^ cells in CD4^+^ and CD8^+^ T cells across the study groups. (A) Representative gating on AIM^+^ cells. (B–D) Percentage frequency of AIM^+^ cells for EBNA-1 (B), BZLF-1 (C), and CMV (D) stimulations, respectively. Centerlines represent medians, with lower and upper boundaries of the boxes representing the first and third quartiles.

Debate continues as to how malaria infection might predispose children from malaria-endemic areas to the EBV-associated cancer eBL. *P. falciparum* is suggested to act as a second stimulus to the B-cell system and by suppression of EBV-specific T-cell surveillance.^5^ Earlier studies have provided support for a malaria-induced suppression of EBV T-cell immunity either through loss of EBV-specific T cells or through functional impairment of circulating T cells. To continue informing on this debate, we set out to examine how an episode of uncomplicated malaria would contribute to the functional impairment of T-cell responses. We investigated the malaria-induced antigen-specific effects on T-cell responses by measuring surface markers for activated T cells (AIM). We evaluated for the expression of known AIM T-cell markers (OX40, CD137, CD25, and CD69) following antigenic stimulation with overlapping peptides to the EBV latent protein EBNA-1, to the EBV lytic protein BZLF1, and to CMV p65. Cells were also treated with the same concentration of DMSO used to stabilize the peptides and as a background control. The representative plots for the AIM^+^ T cells are shown in Fig. 3A. AIM^+^ cells were gated on as CD137^+^CD69^+^ for CD8 T cells and CD25^+^OX40^+^ and CD69^+^OX40^+^ for CD4 T cells. We defined individuals as responders when they had AIM^+^ cells above zero after subtracting an individual’s DMSO background from the different stimulations. Table S2 shows the frequency of responders for the different stimulations. As shown in Fig. 3B and C, there were no significant differences in the frequency of AIM^+^ cells for either CD4^+^ and CD8^+^ T cells following stimulation by EBV EBNA-1 and BZL-F1 peptides. Similarly, we observed no differences in the frequency of AIM^+^ cells for either CD4^+^ and CD8^+^ T cells after stimulation with CMV peptides (Fig. 3D).

### CD4 and CD8 T-cell response is dominated by IL-10–secreting cells in children with uncomplicated *P. falciparum* malaria

**Figure 4.**
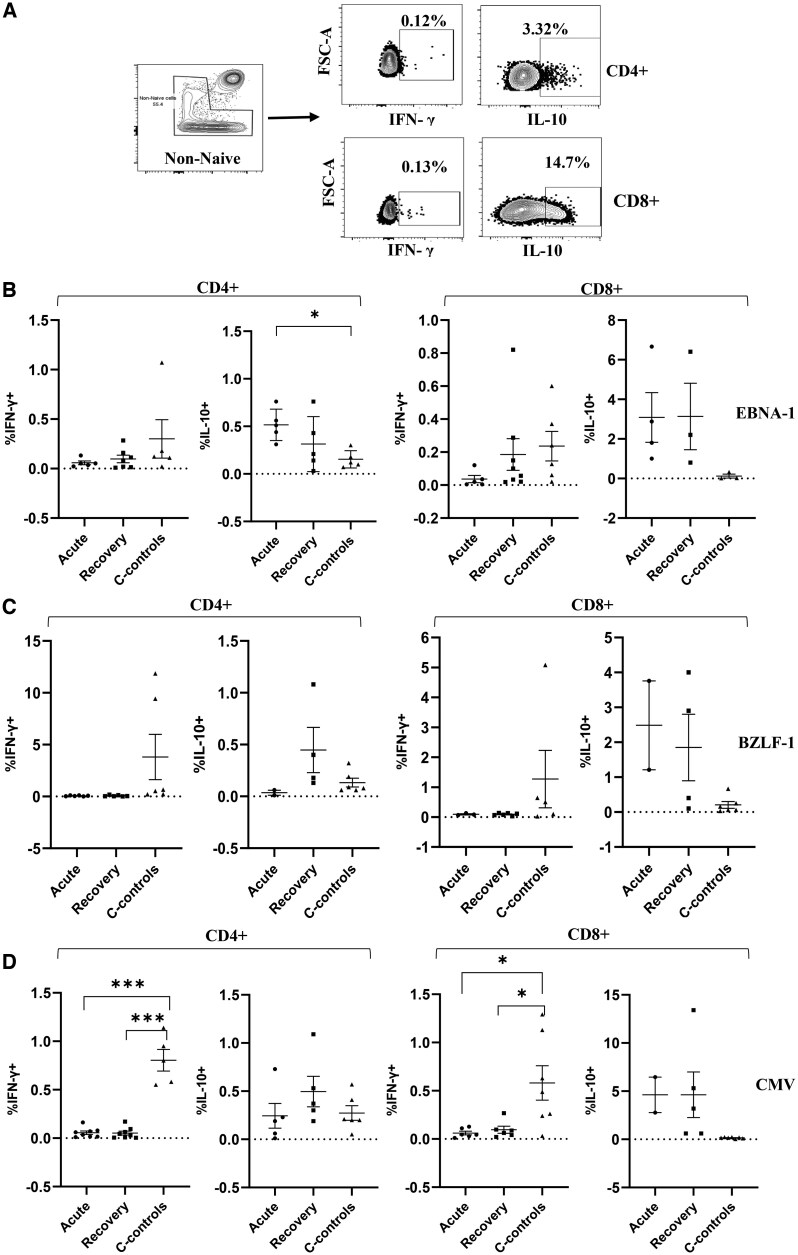
Comparison of cytokine-expressing nonnaïve CD4^+^ and CD8^+^ T cells across the study groups. (A) Representative gating on cells expressing IFN-γ and IL-10. (B–D) Percentage frequency of cytokine-secreting cells for EBNA-1 (B), BZLF-1 (C), and CMV (D) stimulations, respectively. Centerlines represent medians, with lower and upper boundaries of the boxes representing the first and third quartiles, respectively. A *P* value <0.05 was considered significant. *P < 0.01, ***P < 0.0001.

Next, we determined the antigen-specific effects on cytokine secretion within the nonnaïve T-cell compartment following antigenic stimulations with both EBV and CMV peptides. As with the AIM^+^ assay, we also defined our responders by subtracting DMSO as background from an individual’s stimulation. Table S3 shows the number of responders per antigenic stimulation after background subtraction. Remarkably, the frequency of IFN-γ–secreting cells were generally low in children who had experienced uncomplicated malaria compared to community controls for both CD8 and CD4 T cells across different stimulations with significant differences being detectable in CMV-stimulated cells (Fig. 4). On the other hand, even though no significant differences were observed for most of the stimulations except for EBNA-1–stimulated CD4 T cells, children with uncomplicated malaria during active disease and at recovery tended to have a higher frequency of IL-10–secreting cells for both CD8 and CD4 T cells across the different stimulations than community controls (Fig. 4).

## Discussion

*Plasmodium* malaria has been shown to modulate and evade the host immune system.^39^ Even though earlier studies revealed geographic overlap between malaria exposure and eBL and led to the hypothesis that malaria suppresses EBV-specific T-cell immune responses,^5^ the malaria-driven mechanism that underlies T-cell immunity suppression remains obscure. Further, whether the purported immune suppression is more of a global suppression or antigen specific is yet to be elucidated. The present data show that malaria drives immune perturbation across the immune landscape. Consistent with earlier studies, we observed significant changes in the percentage frequencies of CD4^+^ and CD8^+^ T-cell subsets in children with an episode of uncomplicated malaria compared to community controls. The present frequency of AIM^+^ cells was generally comparable across the different stimulations within our study cohort. Our analysis of the cytokine profiles revealed that children with an episode of uncomplicated malaria had reduced frequency of IFN-γ–secreting T cells and upregulated IL-10–producing T-cell subsets. Also, we show that malaria-induced immune T-cell deficit is more of a general suppression rather than EBV specific.

Our baseline analysis focused on EBV and malaria antibody responses in children within the study cohort. The results of this study revealed comparable levels of anti-EBV VCA and EBNA-1 antibody levels in children with uncomplicated malaria during an active disease and at recovery and in community controls. While this result extends in part to previous findings^24^ on EBNA-1 antibody levels in children with eBL and controls, it contradicts earlier studies that reported elevated levels of anti-EBV antibodies in children exposed to malaria relative to controls.^15^^,^^23^^,^^40^ Of note is that children in the study cohort are from a region that is known to experience repeated malaria infections and thus are likely to experience episodes of malaria-induced EBV reactivation^15^^,^^35^—consequently the likely reason for the comparable levels of anti-EBV antibodies within the study cohort. Chronic exposure to *P. falciparum* malaria has been etiologically linked to Burkitt lymphoma.^15^^,^^23^ The elevated levels of anti-MSP-1 antibodies between children with uncomplicated malaria and community controls are in line with other studies that have previously reported elevated preerythrocytic and erythrocytic antigens in chronic malaria exposure and eBL cases.^17^^,^^41^^,^^42^ However, the comparable levels of anti-AMA-1 and anti-CSP contradict the above findings. These differences could again be attributed in part to the study design, in which cases and controls are from malaria-holoendemic regions and experience persistent *P. falciparum* transmission.^43^

The use of flow cytometry AIM assays to analyze immune responses during infections has been useful in identifying key protective and disruptive responses in various disease states.^31^^,^^44^ Here, we leveraged on this assay to understand the antigen-specific T-cell responses in children following uncomplicated *P. falciparum* malaria. This approach allowed for the understanding of the subset-specific cell responses not previously possible with conventional methods such as ELISPOT and regression assays. Within the T-cell lymphocyte subpopulations, children with uncomplicated malaria had altered CD4 and CD8 T-cell subsets compared to community controls. We and others have previously reported altered immunophenotypes of peripheral blood lymphocytes during clinical malaria infection, findings that agree with our current results.^36^^,^^45^^,^^46^ Clinical *P. falciparum* malaria disease is known to induce lymphopenia, a condition that is associated with low lymphocyte count in the peripheral circulation.^47^ The reduced frequency of CD4^+^ and CD8^+^ T-cell subsets and phenotypes during uncomplicated malaria and 4 weeks postrecovery compared to community controls may be due to lymphopenia. Additionally, even though there is a consensus that malaria infection affects lymphocyte count and subpopulations, the degree of the effect is not uniform and varies by disease endemicity, host genetics, and parasite factors. The variations that we see in the T-cell lymphocyte phenotypes within the study population could also be due to this.

Expression of surface markers have been previously used to carry out predictive analysis of the quality and quantity of pathogen-induced T-cell responses.^48^^,^^49^ We analyzed the expression of surface markers that indicate recent activation for CD4 and CD8 T cells (CD69 and CD137 for CD8^+^ T cells and CD69, OX40, and CD25 for CD4^+^ T cells).^32^ We observed comparable frequency of AIM^+^ T cells in children with uncomplicated malaria and community controls among individuals who had responses above the DMSO. Previous studies that characterized T-cell activation profiles during symptomatic malaria infection observed increased expression of activation markers following peptide stimulation,^50^ findings that are not in agreement with our data. While these observations are puzzling, it is worth noting that children with our study cohort are from a malaria-endemic region and are therefore likely to have experienced several episodes of malaria infections prior to the current infection, and therefore likely to have whacked system from the chronic infections. This is supported by the fact that children with uncomplicated malaria during an active disease and at recovery and community controls both had similar levels of malaria antibodies as shown in Fig. 2B, indicative of both recent and past infections. Another mutually not exclusive explanation is the fact that the majority of children with uncomplicated malaria had detectable parasitemia at 4 weeks postrecovery (Fig. S1A); this could possibly explain the lack of differences made between children with uncomplicated malaria during active disease and at recovery.

Evidence of malaria-induced immunosuppression was observed in our data in cytokine secretory profiles, where CD4 and CD8 T-cell responses were dominated by upregulation of IL-10 and downregulation of IFN-γ–secreting cells. We and others have previously reported a deficiency in EBV-specific IFN-γ responses in children with repeated exposure to *P. falciparum* malaria and in patients with confirmed eBL.^13^^,^^24^ Further studies reported impaired T-cell responses and reduced IFN-γ responses in children and adults with an episode of acute clinical *P. falciparum* malaria,^12^ findings that are consistent with our report. Also, we report upregulated expression of IL-10 in children with acute malaria, a finding that is in line with what has been previously reported on the polarization of immune responses by IL-10 in children with acute/repeated exposure to malaria and eBL cases.^38^^,^^51^^,^^52^ The high levels of IL-10 responses in children with acute malaria and significant suppression of IFN-γ–secreting cells witnessed following antigenic stimulation are further indicative of the dysregulation of the immune response induced by malaria.

Of note is that we observed a more global suppression of T-cell immune responses. A model put forward by Liu et al. proposes that repeated infections with one pathogen (eg *Plasmodium*) weaken the total CTL memory, such that established CTL memory to a different pathogen (eg EBV) will be diminished and ultimately collapse.^20^ This model could explain the loss of both EBV and CMV immunity, thus supporting the global suppression that we see during an episode of uncomplicated malaria.

While the findings are intriguing, the study had a couple of limitations that are worth noting. Our malaria cases and community controls were from a malaria-endemic region and thereby likely to have experienced recent or previous malaria infections prior to enrollment in the study. This consequently affected the degree of response following stimulations within the group as reflected by the high number of AIM^+^-expressing cells within the DMSO-treated cells compared to the different stimulations (Fig. 3). This also pointed to the strength of our study design where we compared individuals during an active disease and at recovery making an individual its control. Our sample size among the responders was insufficient to test the association/correlation between the different responses and the *P. falciparum* and EBV loads. Like with other studies, investigating human antigen-specific T cells is quite challenging due to low frequencies of cells captured in peripheral blood samples; the frequency of antigen-specific T cells, for example EBV, was comparable to what has been reported.^51^

In conclusion, our findings provide 2 key suggestions on the role of malaria in the impairment of EBV immunity during an episode of acute *P. falciparum* malaria. First, our data suggest that each episode of clinical malaria is likely to result in the impairment of T-cell activation and shifts in cytokine secretions. Second, we show that malaria-induced T-cell immune deficit is not EBV specific but rather a global suppression since we observed similar responses to both EBV and CMV antigens.

## Supplementary Material

vlaf045_Supplementary_Data

## Data Availability

The data underlying this article will be shared on reasonable request to the corresponding author.
