# Supplementary material for: CD4 and CD8 T-cell response is dominated by IL-10–secreting cells in children with uncomplicated Plasmodium falciparum malaria
Source: Immunohorizons. 2025 Nov 24;9(12):vlaf045. doi: 10.1093/immhor/vlaf045 (PMC12643480; doi:10.1093/immhor/vlaf045)
Supplement: vlaf045_Supplementary_Data [file vlaf045_supplementary_data.pdf]

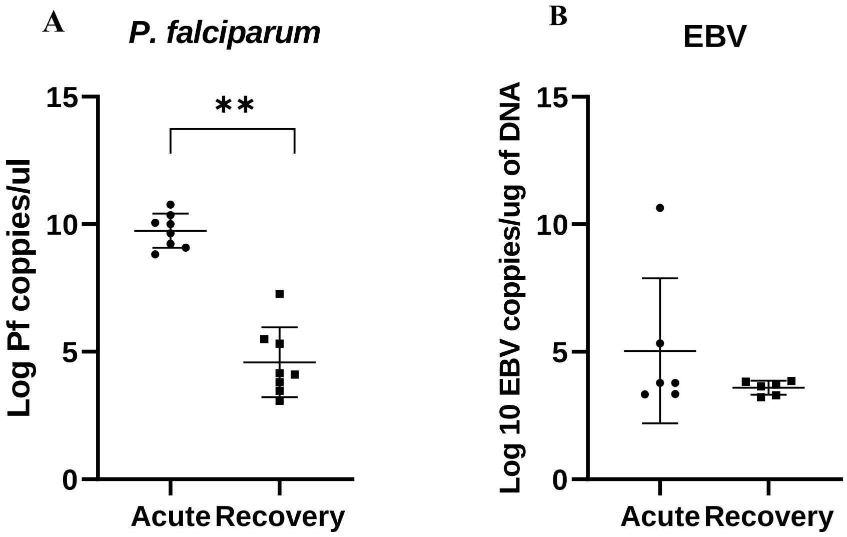

Figure-S1. A. The log *P. falciparum* copies/ ul of blood in subsets of the children during uncomplicated malaria and at 4- weeks recovery. **B**. The log EBV copies/ug of DNA in children during uncomplicated *P. falciparum* malaria infection and at 4- weeks recovery in children that had detectable EBV loads. *P. falciparum* load n=8, EBV load n=6. Wilcoxon test, **p<0.0078.

Table-S1. List of antibodies

Target	Fluorophore	Company
Live Dead	Zombie Aqua/Live dead blue	Biolegend
CD3	AF700	Biolegend
CD4	BV605	Biolegend
CD8	BV650	Biolegend
CD45RA	BV421	Biolegend
CD137	APC	Biolegend
OX40	PE/Cy7	Biolegend
CD69	PE	Biolegend
CCR7	FITC	Biolegend
IL-10	AF 647	Biolegend
IFN-Y	PE-Dazzle 594	Biolegend
CD25	Spark NIR-685	Biolegend

Table-S2. A. Percent frequency of children with responses above DMSO for the individual activation markers.

Antigen stimulation	Activation Marker	T cell Type	Acute	Recovery	C-Controls	Acute vs. Recovery	Acute vs. C-Controls	Recovery vs. C-Controls
EBNA-1	CD25+OX40+	CD4	4/10 (40%)	3/10 (30%)	4/10 (40%)	0.71	1	0.71
	OX40+CD69+		5/10 (50%)	6/10 (60%)	5/10 (50%)	0.76	1	0.76
	CD137+CD69+	CD8	6/10 (60%)	6/10 (60%)	7/10 (70%)	1	0.78	0.78
BZLF-1	CD25+OX40+	CD4	3/10 (30%)	6/10 (60%)	4/10 (40%)	0.32	0.71	0.53
	OX40+CD69+		5/10 (50%)	4/10 (40%)	7/10 (70%)	0.74	0.56	0.37
	CD137+CD69+	CD8	6/10 (60%)	6/10 (60%)	6/10 (60%)	1	1	1
CMV	CD25+OX40+	CD4	3/10 (30%)	5/10 (50%)	4/10 (40%)	0.48	0.71	0.74
	OX40+CD69+		4/10 (40%)	8/10 (80%)	3/10 (30%)	0.25	0.71	0.13
	CD137+CD69+	CD8	10/10 (100%)	7/10 (70%)	7/10 (70%)	0.47	0.47	1

Chi square test

Table-S3. A. Percent frequency of children with responses above DMSO for the individual cytokines.

Cytokine	Antigen Stimulation	T cell Type	Acute	Recovery	C-Controls	Acute vs. Recovery	Acute vs. C-Controls	Recovery vs. C-Controls
IFN- γ	EBNA-1	CD4	5/10 (50%)	7/10 (70%)	5/10 (50%)	0.56	1	0.56
		CD8	5/10 (50%)	8/10 (80%)	6/10 (60%)	0.41	0.76	0.59
IL-10	EBNA-1	CD4	5/10 (50%)	5/10 (50%)	5/10 (50%)	1	1	1
		CD8	4/10 (40%)	3/10 (30%)	3/10 (30%)	0.71	0.71	1
IFN- γ	BZLF-1	CD4	5/10 (50%)	7/10 (70%)	5/10 (50%)	0.56	1	0.56
		CD8	5/10 (50%)	9/10 (90%)	5/10 (50%)	0.29	1	0.29
IL-10	BZLF-1	CD4	5/10 (50%)	5/10 (50%)	5/10 (50%)	1	1	1
		CD8	4/10 (40%)	3/10 (30%)	3/10 (30%)	0.71	0.71	1
IFN- γ	CMV	CD4	8/10 (80%)	8/10 (80%)	5/10 (50%)	1	0.41	0.41
		CD8	6/10 (40%)	6/10 (60%)	7/10 (70%)	1	0.78	0.78
IL-10	CMV	CD4	5/10 (50%)	5/10 (50%)	6/10 (60%)	1	0.76	0.76
		CD8	2/10 (20%)	5/10 (50%)	7/10 (70%)	0.26	0.10	0.56

Chi square test
